# Whorls in the Lung: A Case of Primary Pulmonary Meningioma

**DOI:** 10.7759/cureus.96743

**Published:** 2025-11-13

**Authors:** Yashwanth Suresh Babu, Shahbaz Nazir

**Affiliations:** 1 Internal Medicine, York Hospital, York, GBR; 2 Respiratory Medicine, York Hospital, York, GBR

**Keywords:** extra-cranial meningioma, genetic predisposition, lung tumour mimic, primary pulmonary meningioma, solitary pulmonary nodule

## Abstract

A primary pulmonary meningioma (PPM) is an exceptionally rare, usually benign tumour, with only about 50 cases reported worldwide. We describe a 75-year-old Japanese female patient with a strong family history of gastrointestinal malignancy, in whom a solitary upper-lobe pulmonary nodule was incidentally detected. Radiological evaluation, including Fluorodeoxyglucose-Positron Emission Tomography (FDG-PET), suggested malignancy, but wedge resection and histopathological assessment, supported by immunohistochemistry, confirmed PPM. This case highlights the diagnostic challenge of distinguishing PPM from primary lung carcinoma, reinforces the importance of tissue confirmation and exclusion of intracranial disease, and draws attention to the potential role of genetic or environmental factors in its pathogenesis. By documenting a well-characterized case with an atypical upper-lobe location, this report contributes practical insights for clinicians and pathologists in the diagnosis and management of PPM.

## Introduction

Meningiomas mostly commonly arise from the meningothelial cells of the arachnoid mater. They are the most common primary intracranial neoplasms, accounting for over one-third of primary central nervous system (CNS) tumours [1]. A primary pulmonary meningioma (PPM) is an exceedingly rare ectopic variant that arises within the lung parenchyma; since its first description in the early 1980s, only a few well-documented cases have been reported worldwide [2-6].

PPMs usually present as solitary, well-circumscribed pulmonary nodules, and their radiological appearance is nonspecific. Variable enhancement patterns on contrast-enhanced CT and inconsistent fluorodeoxyglucose (FDG) uptake on positron emission tomography-computed tomography (PET-CT) mean that PPMs can closely mimic primary lung carcinoma or metastatic lesions [7,8]. Histopathological and immunohistochemical analyses are therefore essential for diagnosis. Typical findings include meningothelial whorls with immunoreactivity for epithelial membrane antigen (EMA) and vimentin, frequent progesterone receptor (PR) positivity, and strong somatostatin receptor 2A (SSTR2A) expression, features that help distinguish PPM from metastatic intracranial meningioma and other spindle-cell tumours [5,9].

Although most PPMs behave as benign, slow-growing lesions with excellent outcomes after surgical resection, occasional atypical or malignant variants have been documented [10]. Accurate diagnosis and long-term follow-up are therefore crucial to guide management and surveillance. This case contributes to the limited literature by highlighting the diagnostic challenges posed by the radiologic resemblance of PPM to pulmonary malignancy, underscoring the value of comprehensive histopathological evaluation and adding further clinical and pathological data to refine understanding of this rare entity.

## Case presentation

**Figure 1:**
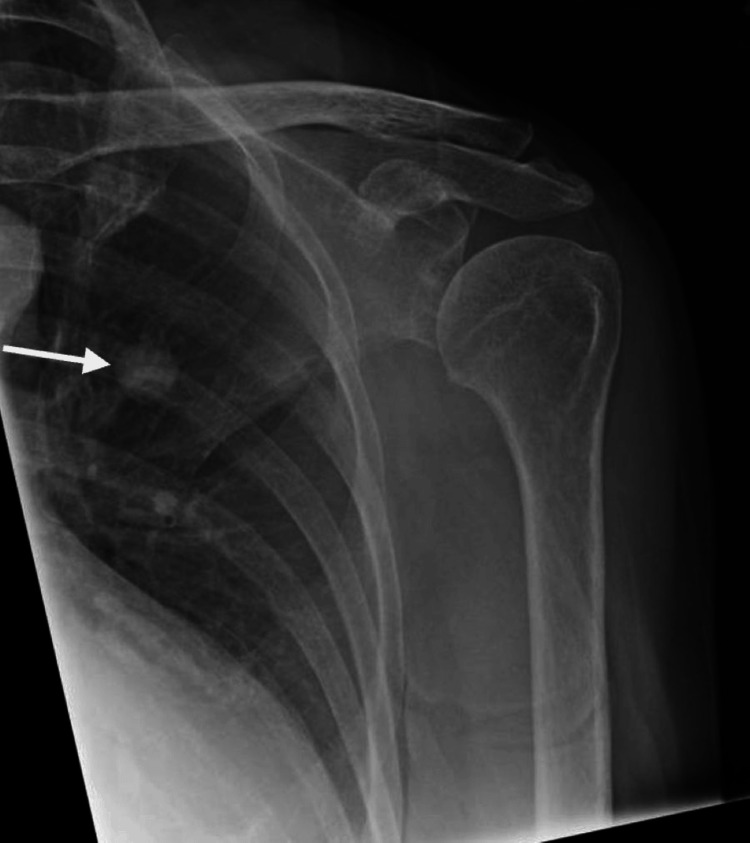
Left shoulder X-ray showing a 16 mm pulmonary nodule (arrow)

A 75-year-old Japanese female patient, a lifelong non-smoker with no history of chronic respiratory disease, was investigated for left shoulder pain in March 2024. She underwent a shoulder X-ray, which revealed an incidental 16 mm nodule in the left lung (Figure 1).

She was asymptomatic from a respiratory perspective, with preserved exercise tolerance, no cough, no dyspnoea, and no constitutional symptoms. Her past medical history was unremarkable, and her family history was notable for gastric cancer in her father and colon cancer in her mother and brother. 

**Figure 2:**
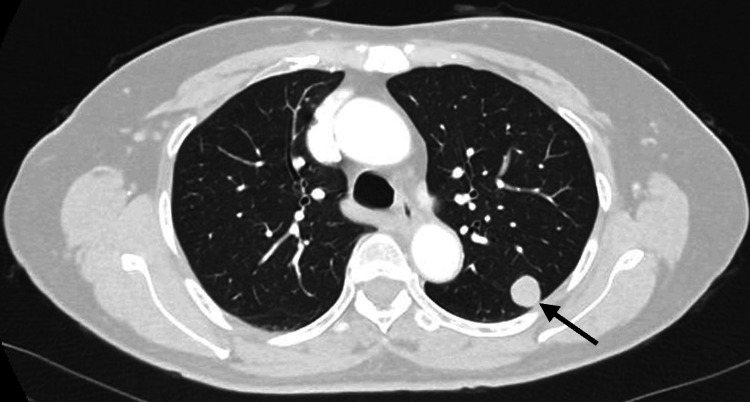
CT scan showing the 16 mm nodule (arrow) in the left lung

Subsequent CT of the thorax, abdomen, and pelvis in April 2024 redemonstrated the solitary nodule suspicious for malignancy, and did not reveal any other masses (Figure 2).

**Figure 3:**
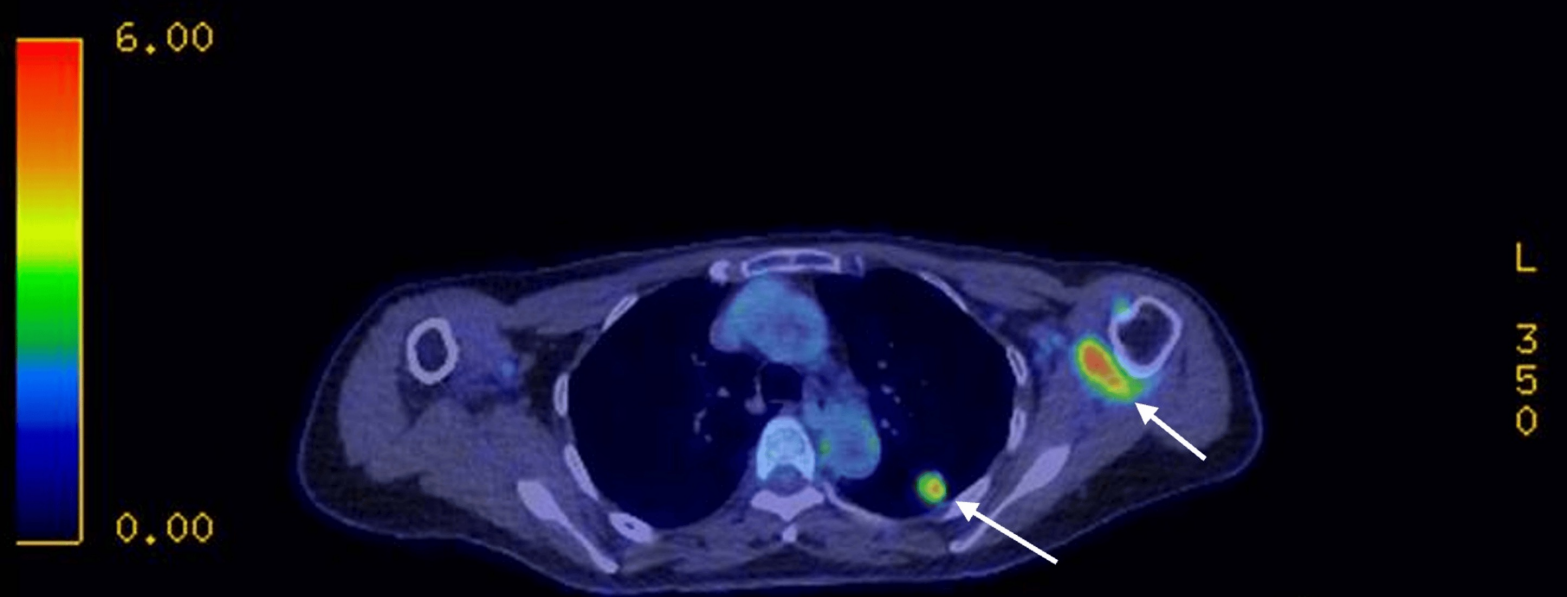
PET-CT showing the 16 mm FDG-avid nodule (SUV max 3.4) and two axillary lymph nodes showing low-grade uptake (SUV 3.0) Left arrow pointing to nodule and right arrow pointing to FDG-avid left axillary lymph nodes; FDG: fluorodeoxyglucose; PET-CT: positron emission tomography-computed tomography.

She then underwent a PET-CT in May 2024 which confirmed a 16 mm FDG-avid, well-circumscribed left upper lobe nodule (SUV max 3.4), with two sub centimetre axillary lymph nodes showing low-grade uptake (SUV 3.0) (Figure 3).

The case was discussed at the lung multidisciplinary team (MDT) and staged radiologically as T2a N0 M0, with tissue diagnosis recommended.

**Figure 4:**
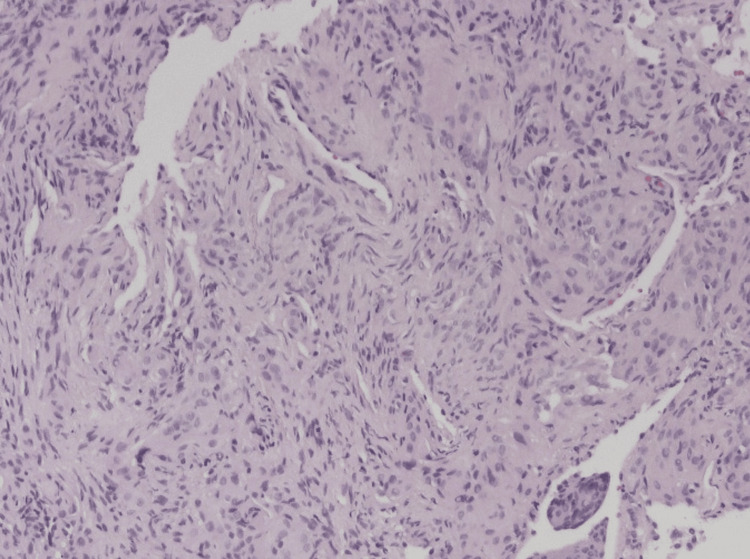
Hematoxylin and eosin (H&E)-stained pathological section (magnification x40), demonstrating uncircumscribed spindle-cell proliferation arranged in whorls and nests

In June 2024, a CT-guided core biopsy was performed. Histology revealed an uncircumscribed spindle-cell proliferation arranged in whorls and nests within the alveolar septa (Figure 4).

The nuclei were oval with frequent intranuclear inclusions, mitoses were absent, and the Ki-67 proliferation index was low. Immunohistochemistry showed positivity for EMA, PR, vimentin, and CD56, with negativity for broad-spectrum cytokeratins, neuroendocrine markers, and myogenic markers. The morphology and immune profile supported a diagnosis of meningioma, with the differential including pulmonary meningothelial-like nodules. Given the size of the lesion (16 mm) and the presence of entrapped nerve tissue, a diagnosis of PPM was favoured. An external neuropathology review in July 2024 confirmed features consistent with meningioma, with no evidence of high-grade behaviour. Given the histological diagnosis, she then underwent an MRI of the brain which did not reveal any intracranial meningioma.

In September 2024, she underwent a wedge excision of the left upper lobe. The pulmonary nodule was completely resected with clear margins. Histology confirmed the diagnosis of PPM, consistent with previous biopsy findings. Three months after the wedge excision, she was reviewed in the clinic and, having remained clinically well, was discharged from routine follow-up.

## Discussion

PPM is an exceptionally rare and predominantly benign tumour that can radiologically and metabolically mimic primary lung carcinoma, as illustrated in this case, where FDG uptake suggested malignancy. Several reports have described similar presentations, with benign PPMs demonstrating moderate-to-high FDG avidity on PET/CT and being initially mistaken for malignant pulmonary lesions [4,7,8]. Radiologically, PPM typically appears as a solitary, well-circumscribed pulmonary nodule. However, this finding is nonspecific and indistinguishable from primary lung carcinoma or solitary metastasis without histological confirmation [2,3].

Histologically, PPMs display spindle or epithelioid meningothelial cells arranged in whorls with intranuclear inclusions, consistent with the features observed in our case. The immunohistochemical profile, positivity for EMA, PR, vimentin, and CD56, and negativity for cytokeratins, neuroendocrine, and myogenic markers, closely parallels that reported in previous literature [5]. Recent studies have also highlighted the diagnostic utility of SSTR2A as a sensitive and specific marker for meningioma, reinforcing its inclusion in diagnostic panels for suspected PPM [9]. These immunophenotypic patterns help distinguish PPM from more common pulmonary tumours such as adenocarcinoma, carcinoid, and solitary fibrous tumours [7].

Demographically, most reported PPMs occur in middle-aged to elderly adults, with a slight female predominance and incidental detection on routine imaging [2,3,7]. Lesions are typically peripheral and well-circumscribed, most often located in the lower lobes, and range in size from 0.6 to 6 cm [7,11]. The present case differs in that the lesion was located in the left upper lobe, highlighting that while lower lobe predilection is common, PPM can arise anywhere within the lung parenchyma. FDG uptake values reported in prior studies vary widely (SUV max 2.0-12.0), underscoring the lack of correlation between metabolic activity and biological behaviour [4,8]. The demographic and radiological features of our case align closely with previously reported cases, with the exception of its upper-lobe location, which broadens the known anatomic distribution of PPM.

A crucial diagnostic step is excluding metastatic intracranial meningioma. In line with prior reports, MRI of the brain was instrumental in our case to confirm the absence of a primary CNS lesion and establish the diagnosis of PPM [3,10]. The absence of necrosis, mitotic activity, and the low Ki-67 proliferation index further supported the benign nature of this tumour, consistent with most previously published cases [2]. This indolent behaviour is exemplified by Satoh and Ishikawa’s 20-year follow-up of a Japanese patient with multiple PPMs, who remained asymptomatic despite slowly growing lesions, confirming the benign biological behaviour of these tumours [12]. Interestingly, our patient is also Japanese, which aligns with several reported cases, although no ethnic predilection has been established; this highlights the importance of documenting demographic patterns in rare tumours. While the prognosis of PPM is generally excellent following surgical excision, isolated cases of malignant transformation or recurrence have been documented, underscoring the need for continued clinical follow-up [10,13,14].

Of additional interest in this case is the patient’s strong family history of gastrointestinal malignancy. The pathogenesis of PPM remains uncertain, with proposed mechanisms including origin from minute pulmonary meningothelial-like nodules, mesenchymal stem cells, or heterotopic arachnoid cell rests [3,7]. Whether genetic, ethnic, or environmental factors contribute to the development of PPM is unknown, as most cases have occurred sporadically in individuals without cancer predisposition syndromes. Given this background, one might ask: is there an unrecognised hereditary or molecular susceptibility that predisposes to meningothelial proliferation outside the CNS? Or is this simply coincidental, reflecting the incidental nature by which most PPMs are discovered?

This case is significant because it contributes to the growing body of literature by illustrating a classic but diagnostically challenging presentation of PPM, a benign lesion with radiological features suggestive of malignancy, and an atypical upper-lobe location. It reinforces the importance of comprehensive histopathological assessment and highlights the ongoing need for molecular characterisation of these rare tumours. Further accumulation of cases with genetic and long-term clinical follow-up will be essential to clarify the mechanisms underlying ectopic meningioma formation and to guide optimal management strategies for future patients.

## Conclusions

A PPM is a rare, benign tumour that can mimic lung malignancy on imaging, making histopathological confirmation essential. This case highlights that PPM can occur in atypical locations, such as the upper lobe, and underscores the importance of considering it in the differential diagnosis of solitary pulmonary nodules. The patient’s ethnicity and family history raise questions about potential genetic or environmental factors. By adding a well-characterized case with detailed radiological and histological features, this study provides practical insights for the diagnosis and management of this rare entity.
